# An original cuproptosis-related genes signature effectively influences the prognosis and immune status of head and neck squamous cell carcinoma

**DOI:** 10.3389/fgene.2022.1084206

**Published:** 2023-01-04

**Authors:** Xiwang Zheng, Chunming Zhang, Defei Zheng, Qingbo Guo, Mijiti Maierhaba, Lingbin Xue, Xianhai Zeng, Yongyan Wu, Wei Gao

**Affiliations:** ^1^ Shanxi Key Laboratory of Otorhinolaryngology Head and Neck Cancer, First Hospital of Shanxi Medical University, Taiyuan, Shanxi, China; ^2^ Shanxi Province Clinical Medical Research Center for Precision Medicine of Head and Neck Cancer, First Hospital of Shanxi Medical University, Taiyuan, Shanxi, China; ^3^ Department of Otolaryngology Head and Neck Surgery, First Hospital of Shanxi Medical University, Taiyuan, Shanxi, China; ^4^ Department of Hematology/Oncology, Children’s Hospital of Soochow University, Suzhou, Jiangsu, China; ^5^ Department of Otolaryngology Head and Neck Surgery, Longgang Otolaryngology Hospital, Shenzhen, Guangdong, China; ^6^ Shenzhen Institute of Otolaryngology and Key Laboratory of Otolaryngology, Longgang Otolaryngology Hospital, Shenzhen, Guangdong, China

**Keywords:** head and neck squamous cell carcinoma, cuproptosis, ISCA2, immune infiltration, prognosis

## Abstract

**Background:** Recently, a non-apoptotic cell death pathway that is dependent on the presence of copper ions was proposed, named as cuproptosis. Cuproptosis have been found to have a strong association with the clinical progression and prognosis of several cancers. Head and neck squamous cell carcinoma (HNSC) are among the most common malignant tumors, with a 5-year relative survival rate ranging between 40% and 50%. The underlying mechanisms and clinical significance of cuproptosis-related genes (CRGs) in HNSC progression have not been clarified.

**Methods:** In this study, expression pattern, biological functions, Immunohistochemistry (IHC), gene variants and immune status were analyzed to investigate the effects of CRGs on HNSC progression. Moreover, a 12-CRGs signature and nomogram were also constructed for prognosis prediction of HNSC.

**Results:** The results revealed that some CRGs were dysregulated, had somatic mutations, and CNV in HNSC tissues. Among them, ISCA2 was found to be upregulated in HNSC and was strongly correlated with the overall survival (OS) of HNSC patients (HR = 1.13 [1.01–1.26], *p*-value = 0.0331). Functionally, CRGs was mainly associated with the TCA cycle, cell cycle, iron-sulfur cluster assembly, p53 signaling pathway, chemical carcinogenesis, and carbon metabolism in cancer. A 12-CRGs signature for predicting the OS was constructed which included, CAT, MTFR1L, OXA1L, POLE, NTHL1, DNA2, ATP7B, ISCA2, GLRX5, NDUFA1, and NDUFB2. This signature showed good prediction performance on the OS (HR = 5.3 [3.4–8.2], *p*-value = 3.4e-13) and disease-specific survival (HR = 6.4 [3.6–11], *p*-value = 2.4e-10). Furthermore, 12-CRGs signature significantly suppressed the activation of CD4^+^ T cells and antigen processing and presentation. Finally, a nomogram based on a 12-CRGs signature and clinical features was constructed which showed a significantly adverse effect on OS (HR = 1.061 [1.042–1.081], *p*-value = 1.6e-10) of HNSC patients.

**Conclusion:** This study reveals the association of CRGs with the progression of HNSC based on multi-omics analysis. The study of CRGs is expected to improve clinical diagnosis, immunotherapeutic responsiveness and prognosis prediction of HNSC.

## Introduction

Copper is a transition metal that is required for essential enzymes and has a key role in cellular metabolism and bioenergy conversion (Kim et al., 2021). Aberrant levels of copper ions have been associated with anemia, cell proliferation and death, metabolic disease and cancer (Ren et al., 2019; Ge et al., 2022). Various forms of cell death, such as apoptosis, necroptosis, pyroptosis and ferroptosis, have been explored in recent years. However, there is limited knowledge on the mechanism of copper overdose-induced cell death (Li et al., 2022). A recent study by Tsvetkov et al. proposed a novel concept of copper-dependent cell death and termed it as cuproptosis (Tsvetkov et al., 2022). The study revealed that copper toxicity was highly associated with mitochondrial activity since key components in mitochondrial metabolism participated in copper ions-induced cell death. The genes coding for key components in copper-dependent cell death have been identified, and may provide a new strategy for the diagnosis, therapy and outcomes prediction of cancers.

Head and neck cancer is one of the most common malignant tumor, accounting for 5.7% of the global cancer mortality (Qin et al., 2022). Head and neck squamous cell carcinoma (HNSC) is the major histological subtype of head and neck cancer, with a 5-year relative survival rate of only 40%–50% (Dai et al., 2020). Analysis of clinical cases revealed that copper levels were significantly correlated with the initiation and progression of HNSC (Ressnerova et al., 2016; Kudva et al., 2021). Although cuproptosis is just a recent concept, there are already studies exploring the ability of cuproptosis-related genes (CRGs) to predict cancer prognosis and their effects on cancer progression (Bian et al., 2022). However, the role CRGs in HNSC is yet to be determined.

In the current study, we comprehensively investigated the effects of CRGs on HNSC progression based on multi-omics analysis, and a multi-genes CRGs signature and nomogram for HNSC prognosis prediction were also constructed. Briefly, we first analyzed the expression profiles of the CRGs and carried out differential expression analysis of CRGs between HNSC and normal tissues. We also analyzed the somatic mutations and copy-number variation (CNV), as wells as protein-protein interactions (PPIs) and biological functions of CRGs. ISCA2 was chosen for further analysis and was found to be up-regulated at the RNA and protein levels, and significantly associated with the prognosis of HNSC. A 12-CRGs gene signature, including CAT, MTFR1L, OXA1L, POLE, NTHL1, DNA2, ATP7B, ISCA2, GLRX5, NDUFA1, NDUFB2, and DLAT, was developed, and exhibited significant ability to predict overall survival (OS) and disease-specific survival (DSS). The 12-CRGs gene signature was also associated with the immune status of HNSC, particularly, the suppression of CD4^+^ T cells activation and antigen processing and presentation. Finally, a nomogram consisting of the 12-CRGs gene signature and clinical features was constructed for clinic utility. In conclusion, the significant association between the expression of CRGs and HNSC progression indicate that CRGs have potential roles as diagnostic, therapeutic and prognostic biomarkers for HNSC.

## Methods and materials

### Data collection

Forty-three cuproptosis-related genes (CRGs) involved in lipoic acid pathway, mitochondria complex I and Fe-S cluster regulation were manually identified from published literature (Tsvetkov et al., 2022). RNA expression and somatic mutation data of 499 head and neck squamous cell carcinoma (HNSC) samples and 45 normal tissues samples were obtained from The Cancer Genome Atlas (TCGA) database (https://portal.gdc.cancer.gov/). The corresponding clinical features of the TCGA samples were downloaded from UCSC Xena database (https://xenabrowser.net/). Firstly, the TCGA samples were first sorted based on the length of overall survival (OS) time, and then pseudo-randomized into two groups in an alternating fashion, designated TCGA-first cohort and TCGA-second cohort. Data for another HNSC cohort consisting of 270 tumor samples and corresponding clinical features (GSE65858) were downloaded from the Gene Expression Omnibus (GEO) database (https://www.ncbi.nlm.nih.gov/gds/). The TCGA-first cohort was used as the training cohort, whereas TCGA-second and GSE65858 were used as the validation cohorts. The clinical features of the three cohorts are summarized in the Supplementary Table S1.

### Expression pattern analysis

The expression profile of 43 CRGs was visualized using a heat map that was generated using the pheatmap package (v1.0.12) in R (v4.1.0) based on unsupervised clustering. Principal Component Analysis (PCA) was used to determine the ability of the 43 CRGs to discriminate between HNSC and normal tissues, and was carried out using the stats (v4.1.0) and pca3d (v0.10.2) packages in R. The correlation amongst the 43 CRGs was analyzed using the stats and corrplot (v0.92) packages in R. The boxplot showing the expression of the 43 CRGs was generated using the ggpubr package in R (v0.4.0), while Student’s t-test was used for statistical analysis between HNSC and normal tissues.

### Somatic mutation analysis

The maftools (v2.8.05) package in R was used to visualize and analyze the somatic mutation of the 43 CRGs based on Mutation Annotation Format (MAF) from TCGA database. In addition, GISTIC2 (v2.0.23) for Linux (Ubuntu; v20.04.3) was utilized to analyze the somatic copy-number variations (CNV) of HNSC tissues.

### Protein-protein intersections and biological function analysis

Protein-protein intersections (PPIs) among the 43 CRGs were built in the STRING database (v11.5; https://string-db.org/). In addition, PPI networks for 479 additional proteins together with the 43 CRGs were also generated from the STRING database, and visualized using Cytoscape (v3.7.1). Functional enrichment analysis including Gene Ontology (GO) and KEGG analysis of CRGs was implemented using R package clusterProfiler (v4.0.5) and the results visualized using the ggplot2 package (v3.3.6) in R.

### Cox regression and survival analysis

First, univariate Cox regression analysis was used to determine the effects of the 43 CRGs and clinical features on survival of the patients. Next, the risk factors that showed significant effect on survival in the univariate analysis including CRGs ISCA2, gender, age, pathologic stage and alcohol history, were used for multivariate Cox regression analysis. Cox regression analysis was carried out using the survival (v3.3–1) package in R, while the results were visualized using the forestplot (v2.0.1) package. Kaplan-Meier estimate curves were used for estimation of survival probability over 5 years and were generated and visualized using survival and survminer (v0.4.9) packages, respectively. Wald test in Cox regression and log-rank test in Kaplan-Meier estimate were applied to assess the statistical differences among different groups. OS probability of ISCA2 was also verified in all TCGA HNSC samples using GEPIA2 webserver (http://gepia2.cancer-pku.cn/).

### Immunohistochemistry analysis

Immunohistochemistry (IHC) was used to validate the expression of ISCA2 at proteome level. IHC images of ISCA2 in HNSC and normal tissues were obtained from the Human Protein Atlas (https://www.proteinatlas.org/). The ISCA2 antibody used for immunohistochemistry was HPA030492 (Atlas antibodies, Bromma, Sweden).

### Least absolute shrinkage and selection operator analysis and construction of CRGs risk score model

Least absolute shrinkage and selection operator (LASSO) was used to shrink the data dimensionality, extract representative features and build the CRGs risk-score model. LASSO analysis was carried out using the glmnt (v4.1–4) package in R. We then calculated the risk score for each individual using the following formula: $risk\;score=\sum_{i=1}^{n}\left({co}_{CRGsi}\times {ex}_{CRGsi}\right)$ , where ${ex}_{CRGsi}$ is expression level of the risk factor CRGsi ; ${co}_{CRGsi}$ is the coefficient of the risk factor CRGsi ; n is the number of the risk factors in the CRGs risk score model. A Kaplan-Meier curve was used to validate the predictive ability of upper risk score model. The time-independent Receiver Operating Characteristic (ROC) curve and area under time dependent ROC curve (AUC) were utilized to evaluate the sensitivity and specificity of the risk score model. The calculation and visualization of the time-independent ROC was done using the timeROC (v0.4) and ggplot2 packages, respectively.

### Validation of CRGs expression in tumor tissues

To validate the expression levels of genes in CRGs risk score model, expression of selected genes in 57 paired laryngeal squamous cell carcinoma (LSCC, one of the most common HNSC) and matched adjacent normal mucosa (ANM) tissues were analyzed using RNA sequencing technology and bioinformatic analysis, of which detailed workflows and methods were described in our previous study (Wu et al., 2020).

### The effects of CRGs risk score model on immune infiltration

The 28 immune cell types and the 21 immune-related pathways analyzed in this study were identified from the tumor and immune system interaction database (TISIDB; http://cis.hku.hk/TISIDB) and the Kyoto Encyclopedia of Genes and Genomes (KEGG; https://www.kegg.jp/), respectively. The single sample gene set enrichment analysis (ssGSEA) was employed to estimate the immune infiltration levels based on HNSC expression profile. Enrichment scores of ssGSEA were calculated using the GSVA (v1.40.1) package in R, and Student’s t-test was used to compare immune infiltration level and CRGs risk score model.

### Nomogram construction

A nomogram that combined the CRGs risk score model and clinical features was built using the rms (v6.3–0) package in R. The nomogram was used to predict the 1-, 2-, three- and 5-year OS probability. The calibration curve used to evaluate the prediction ability of nomogram was generated using the rms package in R. Furthermore, decision curve analysis was employed to assess the clinical utility of the nomogram, and was visualized using the rmda (v1.6) package in R. The nomogram score of each patient was calculated, and the patients were divided into high risk and low risk groups based on the median nomogram score. Cox regression analysis and Kaplan-Meier estimate curves were utilized to estimate the effects of nomogram risk score on 5-year OS.

### Statistical analysis

All statistical analyses were performed in R program in this study. Student’s t-test was used for statistical analysis of CRGs expression between HNSC and normal tissues. Wald test was used for assessing the statistical significance in Cox regression analysis. Significance was tested using the Log-rank test in Kaplan-Meier estimate. Over-representation test that is the default testing method in clusterProfiler package in R language was used to significant analysis in enrichment analysis. Unless the significant cutoff was specifically indicated, it was assumed to be *p-value* < 0.05.

## Results

### The expression patterns of CRGs was significantly correlated with HNSC

**FIGURE 1:**
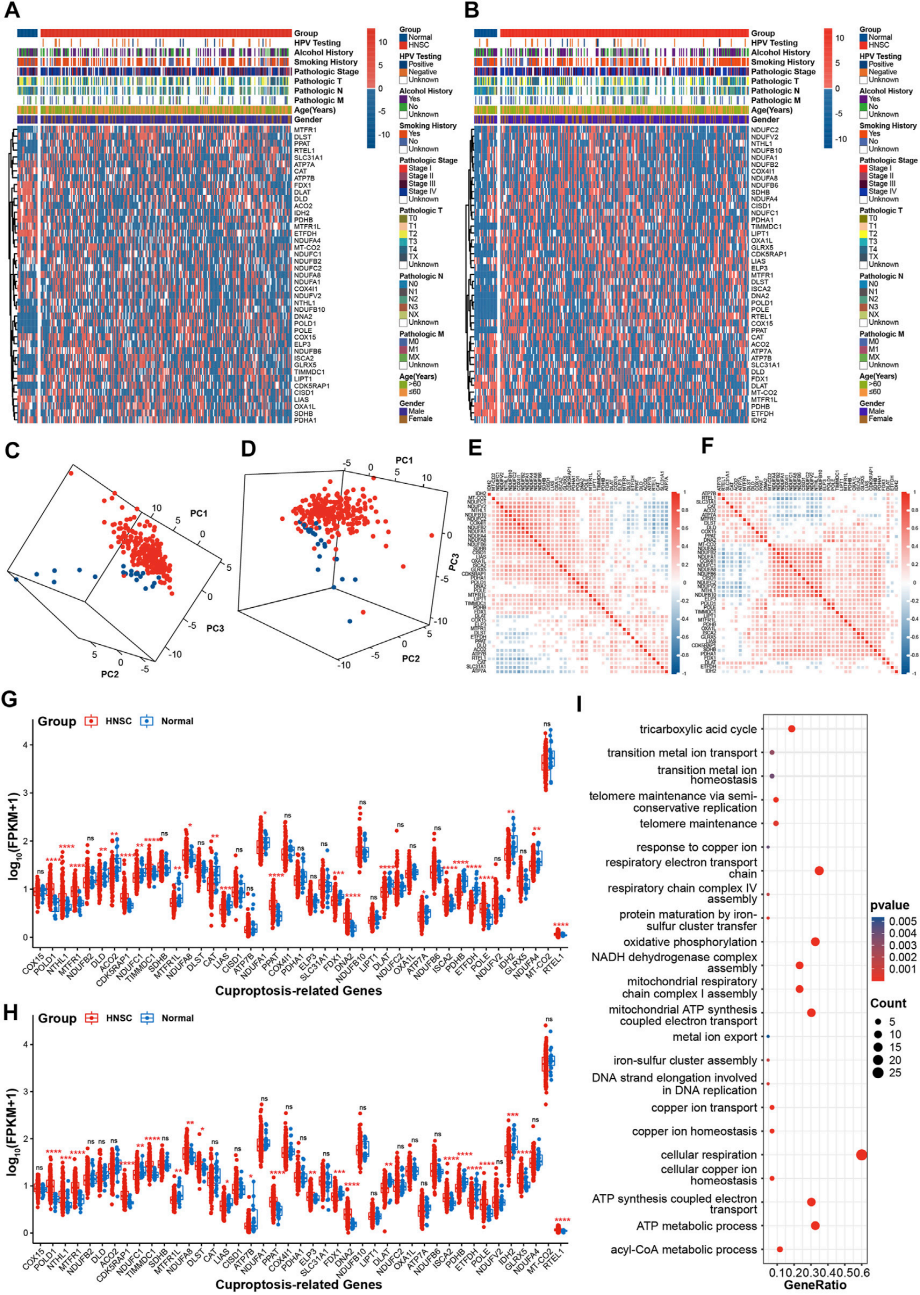
Expression patterns of CRGs in HNSC **(A**,**B)** The heatmap of CRGs in TCGA-first **(A)** and TCGA-second **(B)** cohort **(C**, **D)** 3D PCA plots of CRGs in TCGA-first **(C)** and TCGA-second **(D)** cohorts, red: HNSC tissues; blue: normal tissues **(E,F)** The correlations analysis of CRGs in TCGA-first **(E)** and TCGA-second **(F)** cohorts **(G–H)** Boxplot showing of CRGs expression in TCGA-first **(G)** and TCGA-second **(H)** cohorts **(I)** Enrichment analysis of GO biological process terms. *p-value < 0.05; **p-value < 0.01; ***p-value < 0.001; ****p-value < 0.0001.

A total of 43 CRGs were manually identified and used to investigate the effects of cuproptosis in HNSC in current study (Supplementary Table S2). The workflow of the bioinformatics analysis is shown in Supplementary Figure S1. The heat map revealed that there was significant difference in the expression patterns of CRGs between HNSC and normal tissues (Figures 1A, B). Moreover, the 3D plot of PCA showed that the expression pattern of CRGs in HNSC was distinct from that in normal tissues (Figures 1C,D). To investigate the internal correlation of CRGs, the expression correlation analysis was performed. Figures 1E, F indicate that internal correlation of CRGs was similar in both TCGA-first and TCGA-second cohort. Especially, genes in Fe-S cluster complex I were highly correlated with each other. Results of statistical analysis showed that eleven CRGs (POLD1, NTHL1, MTFR1, CDK5RAP1, TIMMDC1, NDUFA8, PPAT, DNA2, ISCA2, POLE, RTEL1) were upregulated in HNSC compared with normal tissues, while eight genes (NDUFC1, MTFR1L, LIAS, FDX1, DLAT, PDHB, ETFDH, IDH2) were downregulated expression in HNSC tissues in both TCGA-first and TCGA-second cohorts (Figures 1G, H). Moreover, statistical analysis based on paired samples extracted from all TCGA HNSC samples also validated the expression difference of the above nineteen CRGs between HNSC and normal tissues (Supplementary Figure S2). In addition, GO enrichment analysis revealed that the CRGs were mainly enriched in the tricarboxylic acid cycle, iron-sulfur cluster assembly, copper ion response/transport/homeostasis process and other pathways related to respiratory metabolism (Figure 1I), which was consistent with the results of the previous study (Tsvetkov et al., 2022).

### CRGs had somatic mutations in HNSC

**FIGURE 2:**
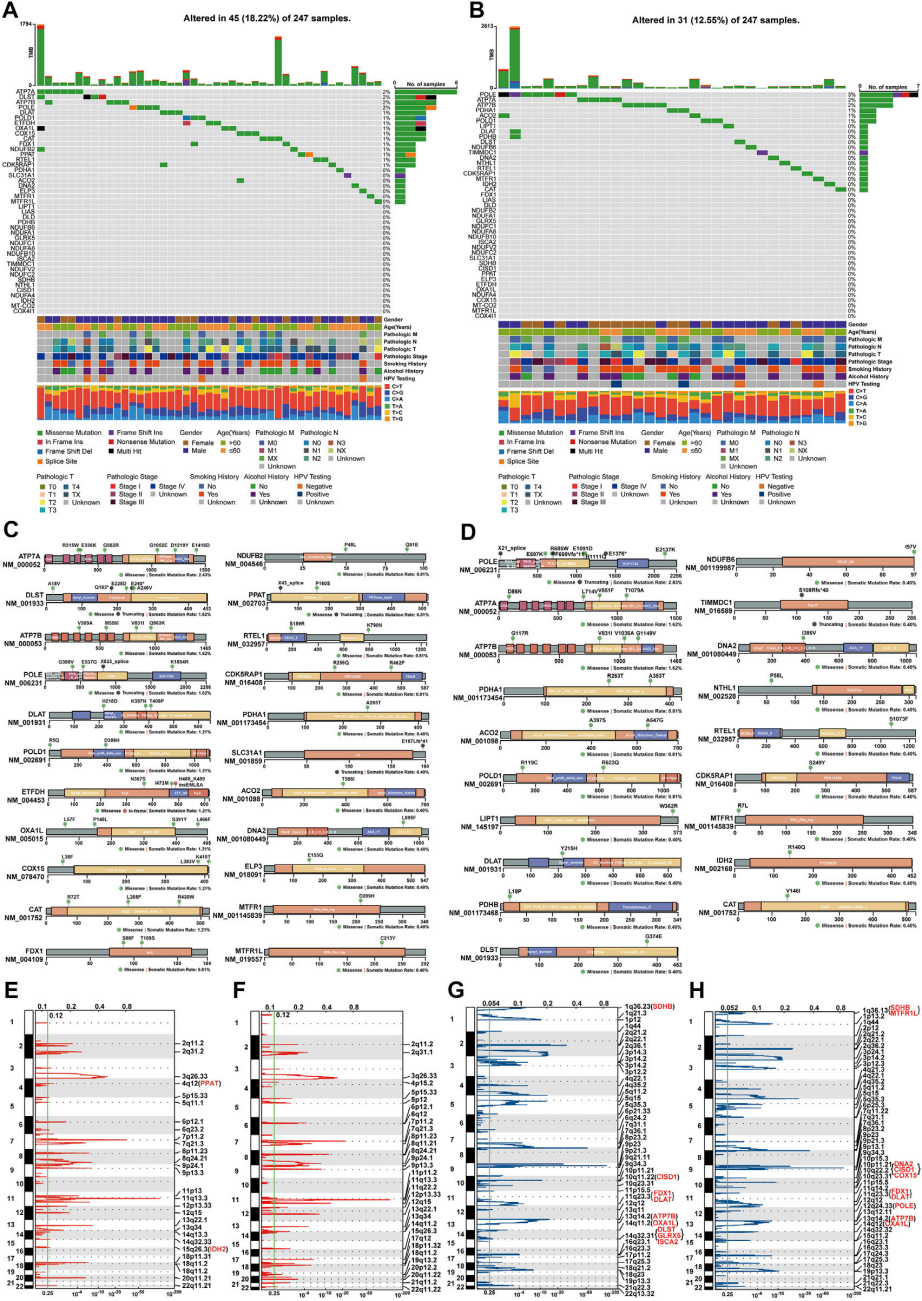
Analysis of gene variants of CRGs in HNSC **(A**,**B)** Mutational oncoplot of CRGs in TCGA-first **(A)** and TCGA-second **(B)** cohorts **(C**,**D)** Changes in proteins structure of CRGs due to somatic mutations in TCGA-first **(C)** and TCGA-second **(D)** cohorts **(E**,**F)** Amplification of chromosomal sub-bands of CRGs in TCGA-first **(E)** and TCGA-second **(F)** cohort **(G**,**H)** Chromosomal deletion of sub-bonds of CRGs in TCGA-first **(G)** and TCGA-second **(H)** cohorts.

To investigate the mutational patterns of CRGs in HNSC, MAF files of HNSC samples were used for somatic mutation analysis. Oncoplots showed that thirteen genes (MTFR1, DNA2, ACO2, PDHA1, CDK5RAP1, RTEL1, CAT, POLD1, DLAT, POLE, ATP7B, DLST, ATP7A) had at least one somatic mutation in both TCGA-first and TCGA-second cohorts (Figures 2A, B). The altered ratios in TCGA-first and TCGA-second cohorts were 18.22% (45 of 247 samples) and 12.55% (31 of 247 samples), respectively. Further, the mutational lollipop plots indicated that the gene mutations affected the spatial structure of proteins (Figures 2C, D). Since CNV affects gene expression (Fang et al., 2016), we also investigated the amplifications and deletions of genes in CRGs. The heat map of raw CNV number indicated that TCGA-first and TCGA-second cohorts had a similar CNV landscape (Supplementary Figures S3A, B). Figures 2E, F show the somatic chromosomal amplifications of genes in CRGs. Deletion analysis revealed that there were clear copy number deletions in the chromosomal sub-bands where SDHB, CISD1, FDX1, DLAT ATP7B, and OXA1L are located (Figures 2G, H). It should be noted that FDX1 and DLAT had chromosomal deletions as well as downregulated expression, indicating that chromosomal deletions of FDX1 and DLAT might influence their expression levels.

### Construction of PPIs networks and functional enrichment analysis

**FIGURE 3:**
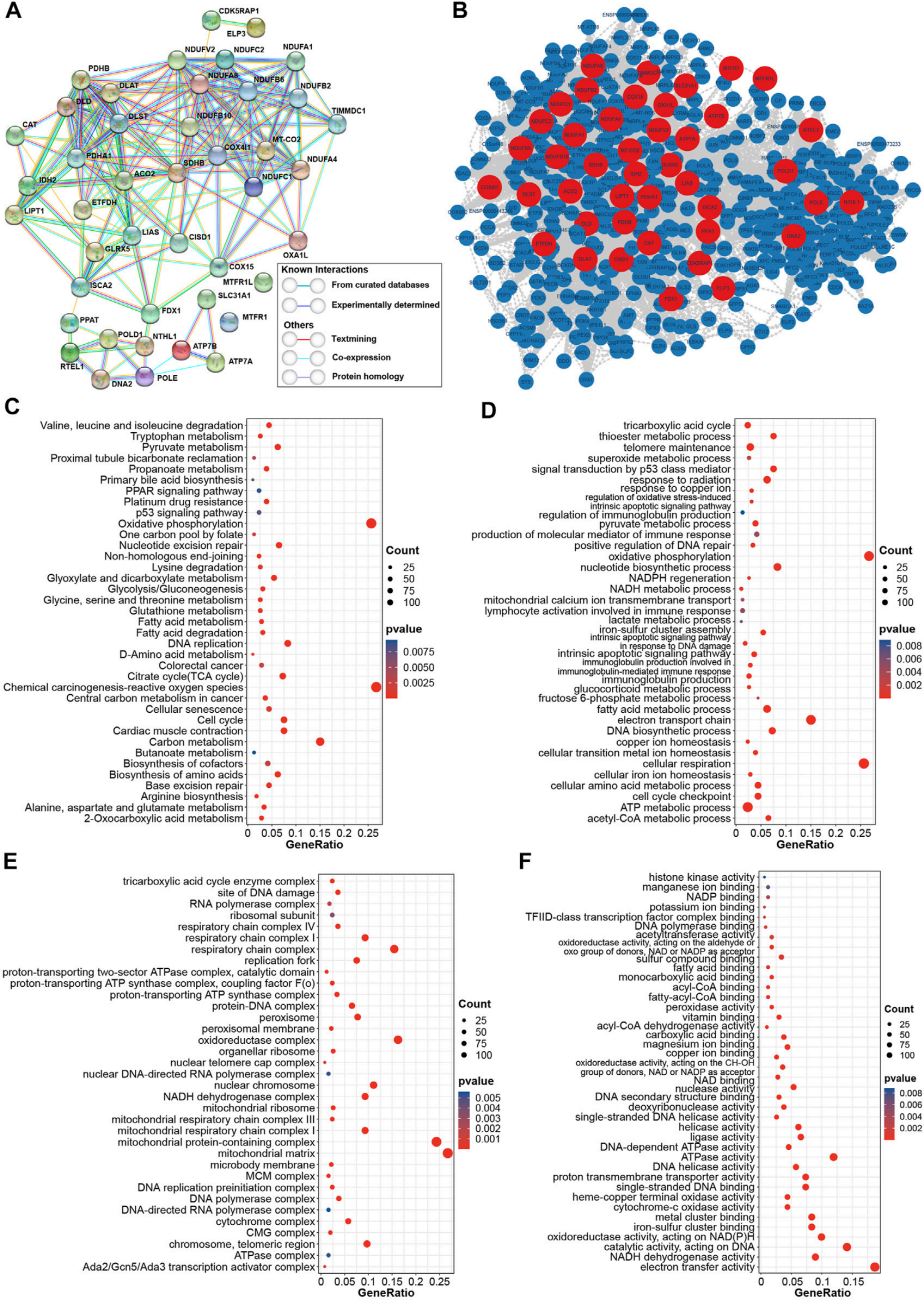
Construction of PPIs network and functional enrichment analysis **(A)** Internal PPIs network of CRGs obtained from STRING database **(B)** PPIs network of 522 CRPs from the STRING database, red: proteins coded by CRGs; blue: additional proteins obtained from the STRING database **(C)** KEGG enrichment analysis of CRPs **(D)** Enrichment analysis for CRPs based on GO biological process terms **(E)** Enrichment analysis for CRPs based on GO cellular component terms **(F)** Enrichment analysis for CRPs based on GO biological function terms.

To study the intrinsic interactions amongst CRGs, the PPIs network was built in STRING database. The PPIs network in Figure 3A revealed the potential connection among CRGs based on diverse interactions. Proteins in lipoic acid pathway and Fe-S cluster complex I were highly respective correlation. Further, 479 additional proteins that interacted with CRGs were also obtained from the STRING database, and their PPIs network are shown in Supplementary Table S3 and Figure 3B. The up- and downstream collaboration of various proteins in a signaling pathway is one of the important PPIs ways. All cuproptosis-related proteins (CRPs) included proteins coded by 43 CRGs and 479 additional proteins were subsequently utilized for functional enrichment analysis. KEGG analysis revealed that CRPs were mainly enriched in p53 signaling pathway, TCA cycle, cell cycle, glycolysis/gluconeogenesis, chemical carcinogenesis, carbon metabolism in cancer and other diseases and metabolism pathways (Figure 3C). Similarly, GO of biological process analysis showed that CRPs were enriched in p53-related pathway, TCA cycle and cell cycle pathway. In addition, CRPs were also enriched in iron ion regulation, iron-sulfur cluster assembly, immune-related regulation, synthetic and repair of DNA and energy metabolism signaling pathway in biological process analysis (Figure 3D). Figures 3E,F show the enrichment results of CRPs in the cellular component terms and molecular function terms of GO analysis. The similar biological functions, such as TCA cycle, mitochondrial components, DNA polymerase complex, ATPase activity, DNA helicase activity and iron-sulfur cluster binding, have validated the analysis process of GO biological process terms.

### ISCA2 was upregulated and strongly associated with prognosis of HNSC

**FIGURE 4:**
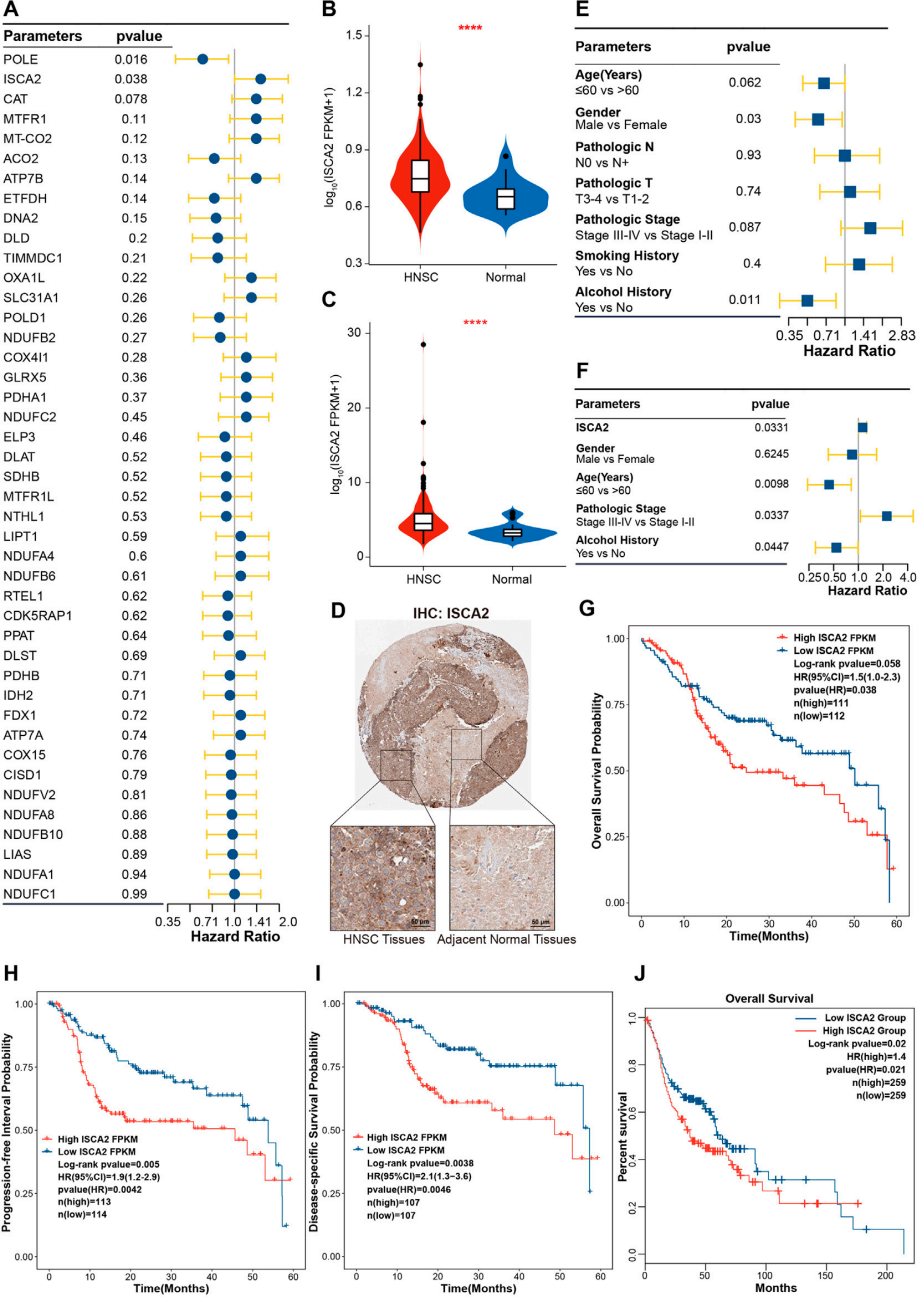
ISCA2 was upregulated and significantly correlated with HNSC progression **(A)** Univariate Cox regression analysis of CRGs **(B**,**C)** Boxplot showing ISCA2 expression in TCGA-first **(B)** and TCGA-second **(C)** cohorts **(D)** Validation of ISCA2 expression by IHC in HNSC and normal tissues **(E)** Univariate Cox regression analysis of clinical features **(F)** Multivariate Cox regression analysis of risk factors consisting of ISCA2, gender, age pathologic stage, and alcohol history **(G–I)** Kaplan-Meier curve showing the association of ISCA2 expression with the 5-year OS **(G)**, PFI **(H)**, and DSS **(I,J)** Association of ISCA2 with the OS in all TCGA HNSC samples as verified GEPIA2 webserver. ****p-value < 0.0001.

To investigate the prognostic effects of CRGs in HNSC, univariate Cox regression was used to analyze the correlation between the single gene expression of CRGs and the OS of HNSC. Figure 4A shows that ISCA2 and POLE were significantly correlated with OS of HNSC, with ISCA2 being a poor prognostic factor for HNSC OS (Hazard ratio (HR) = 1.5 [1.0–2.3], *p*-value = 0.038). Expression of ISCA2 was upregulated in HNSC at the transcriptome level (Figures 1G, H, 4B,C). Moreover, IHC analysis was utilized to validate the expression of ISCA2 in proteome. IHC results in the Human Protein Atlas database showed that ISCA2 was lowly expressed in normal tissues, while high level of ISCA2 was observed in HNSC tissues (Figure 4D). We also analyzed the effects of clinical features on the prognosis. Results of Cox regression analysis indicated that age, gender, pathologic stage and alcohol history were risk factors (Figure 4E). We further investigated the synergistic effects of ISCA2 and clinical features using multivariate Cox regression analysis (Figure 4F). In the multivariate Cox model, ISCA2 (HR = 1.13 [1.01–1.26], *p*-value = 0.0331), age (HR = 0.44 [0.24–0.82], *p*-value = 0.0098), pathologic stage (HR = 2.23 [1.06–4.66], *p*-value = 0.0337) and alcohol history (HR = 0.54 [0.3–0.99], *p*-value = 0.0447) demonstrated significant effects on the OS of HNSC. The Kaplan-Meier method was employed to validate the effects of ISCA2 in different prognosis. Results in Figures 4G–I indicate that ISCA2 had significant effects on the probability of OS, progression-free interval (PFI; HR = 1.9 [1.2–2.9], *p*-value = 0.0042) and diseases-specific survival (DSS; HR = 2.1 [1.3–3.6], *p*-value = 0.0046) in the training cohort. The effect trends of ISCA2 on prognosis in TCGA-first cohort was validated in the TCGA-second and GSE65858 cohorts (Supplementary Figure S4). In addition, effects of ISCA2 on OS probability of all TCGA HNSC samples was also verified in GEPIA2 webserver (Figure 4J).

### Construction of a 12-CRGs signature for OS prediction of HNSC

**FIGURE 5:**
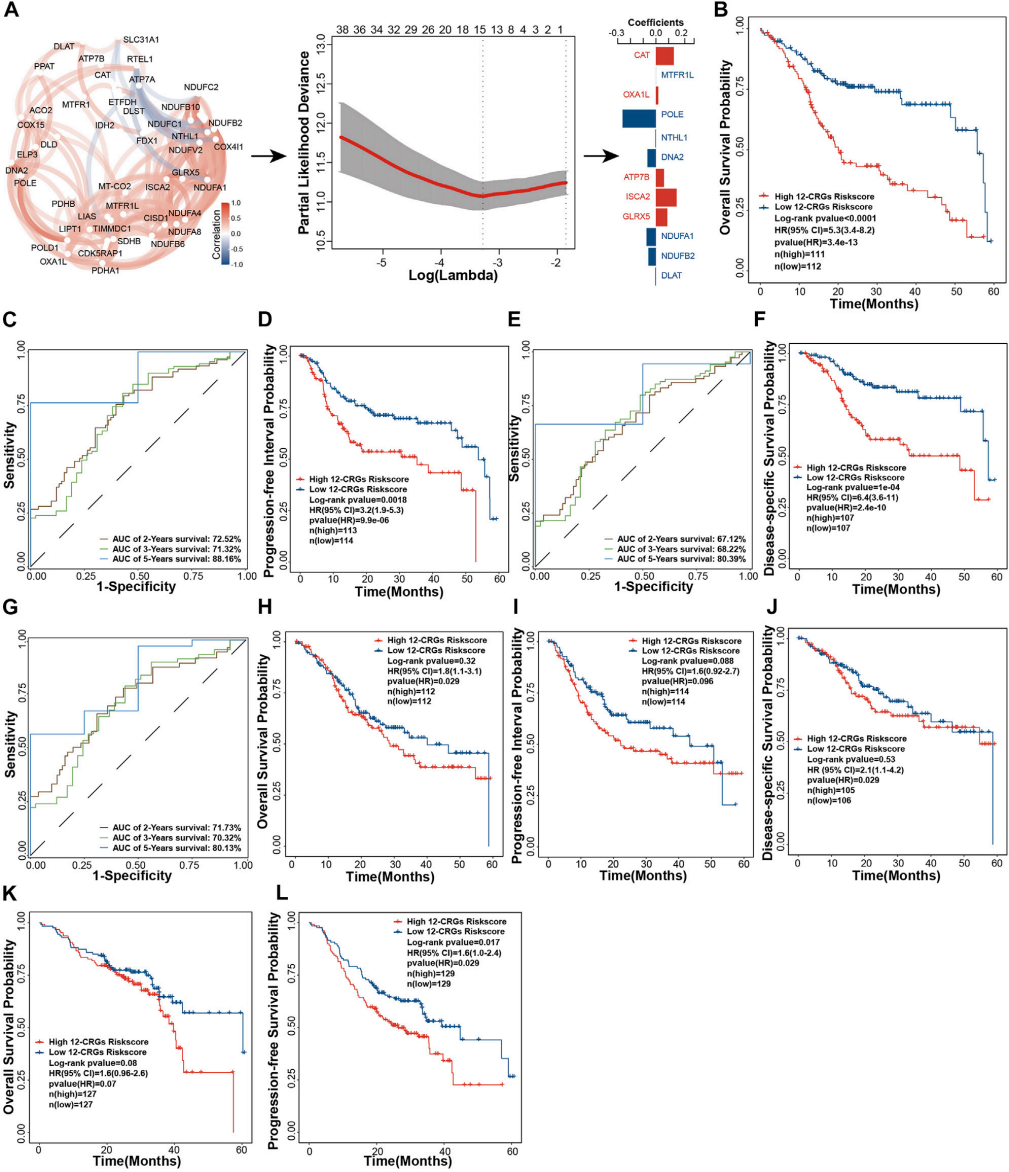
Construction and validation of 12-CRGs signature for OS prediction of HNSC **(A)** Schematic diagram of 12-CRGs signature construction **(B)** Kaplan-Meier curve showing the OS predicted by the 12-CRGs signature over 5 years **(C)** AUC values of the time-dependent ROC curves showing the predicted ability of the 12-CRGs signature for OS in 2-, 3- and 5-year **(D)** Kaplan-Meier curve showing the PPI predicted by the 12-CRGs signature over 5 years **(E)** AUC values of the time-dependent ROC curves showing the predicted ability of the 12-CRGs signature for PPI in 2-, 3- and 5-year **(F)** Kaplan-Meier curve showing the DSS predicted by the 12-CRGs signature over 5 years **(G)** AUC values of the time-dependent ROC curves showing the predicted ability of the 12-CRGs signature for DSS in 2-, 3- and 5-year **(H–J)** Kaplan-Meier curve of OS **(H)**, PPI **(I)** and DSS **(J)** predicted by 12-CRGs signature over 5 years in TCGA-second cohort **(K,L)** Kaplan-Meier curve of OS **(K)** and progression-free survival **(L)** predicted by 12-CRGs signature over 5 years in GSE65858 cohort.

Multi-gene-combination signatures have a higher sensitivity than the single index in prediction of clinical outcomes (Ekanem et al., 2019). Forty-three CRGs were used to construct a multi-genes signature based on LASSO algorithm. A 12-CRGs signature, including CAT, MTFR1L, OXA1L, POLE, NTHL1, DNA2, ATP7B, ISCA2, GLRX5, NDUFA1, NDUFB2, and DLAT, was finally developed (Figure 5A). Risk scores based on the expression levels of the 12-CRGs signature for each patient were calculated utilizing follow formula: risk scores = 0.1407 × ${ex}_{CAT}$ + −0.0002 × ${ex}_{MTFR1L}$ + 0.0179 × ${ex}_{OXA1L}$ + −0.2615 × ${ex}_{POLE}$ + −0.0043 × ${ex}_{NTHL1}$ + −0.0667 × ${ex}_{DNA2}$ + 0.0628 × ${ex}_{ATP7B}$ + 0.1622 × ${ex}_{ISCA2}$ + 0.0890 × ${ex}_{GLRX5}$ + −0.0733 × ${ex}_{NDUFA1}$ + −0.0589 × ${ex}_{NDUFB2}$ + −0.0045 × ${ex}_{DLAT}$ . In TCGA-first cohort, patients were divided into high and low risk groups based on the median risk score of the 12-CRGs signature. Figures 5B, D,F show that patients in the high-risk group had the more adverse outcomes of OS (HR = 5.3 [3.4–8.2], *p*-value = 3.4e-13), PFI (HR = 3.2 [1.9–5.3], *p*-value = 9.9e-06), and DSS (HR = 6.4 [3.6–11], *p*-value = 2.4e-10). The AUC of time dependent ROC was used to evaluate the sensitivity and specificity of 12-CRGs risk scores in prognostic prediction. The AUC for OS, PFI and DSS was 72.52%, 71.32% and 88.16%, 67.12%, 68.22% and 80.39, 71.73, 70.32% and 80.13% in the time period of 2-, three- and 5-year, respectively (Figures 5C,E,G). Unsurprisingly, 12-CRGs signature also had the significant effects on the probability of OS (HR = 1.8 [1.1–3.1], *p*-value = 0.029), and DSS (HR = 2.1 [1.1–4.2], *p*-value = 0.029) in TCGA-second cohort (Figures 5H,J). In addition, although its effect on PFI in TCGA-second cohort was not significantly different (HR = 1.6 [0.92–2.7], *p*-value = 0.096), the 12-CRGs risk scores have showed a tendency toward being a risk factor based on the clear separation of probability curves between high and low risk group (Figure 5I). Further, the trend of 12-CRGs as the risk factor was also validated in the prognosis analysis of GSE65858 cohort that included OS (HR = 1.6 [0.96–32.6], *p*-value = 0.07) and progression-free survival (PFS; HR = 1.6 [1.0–2.4], *p*-value = 0.029) analysis (Figures 5K,L).

**FIGURE 6:**
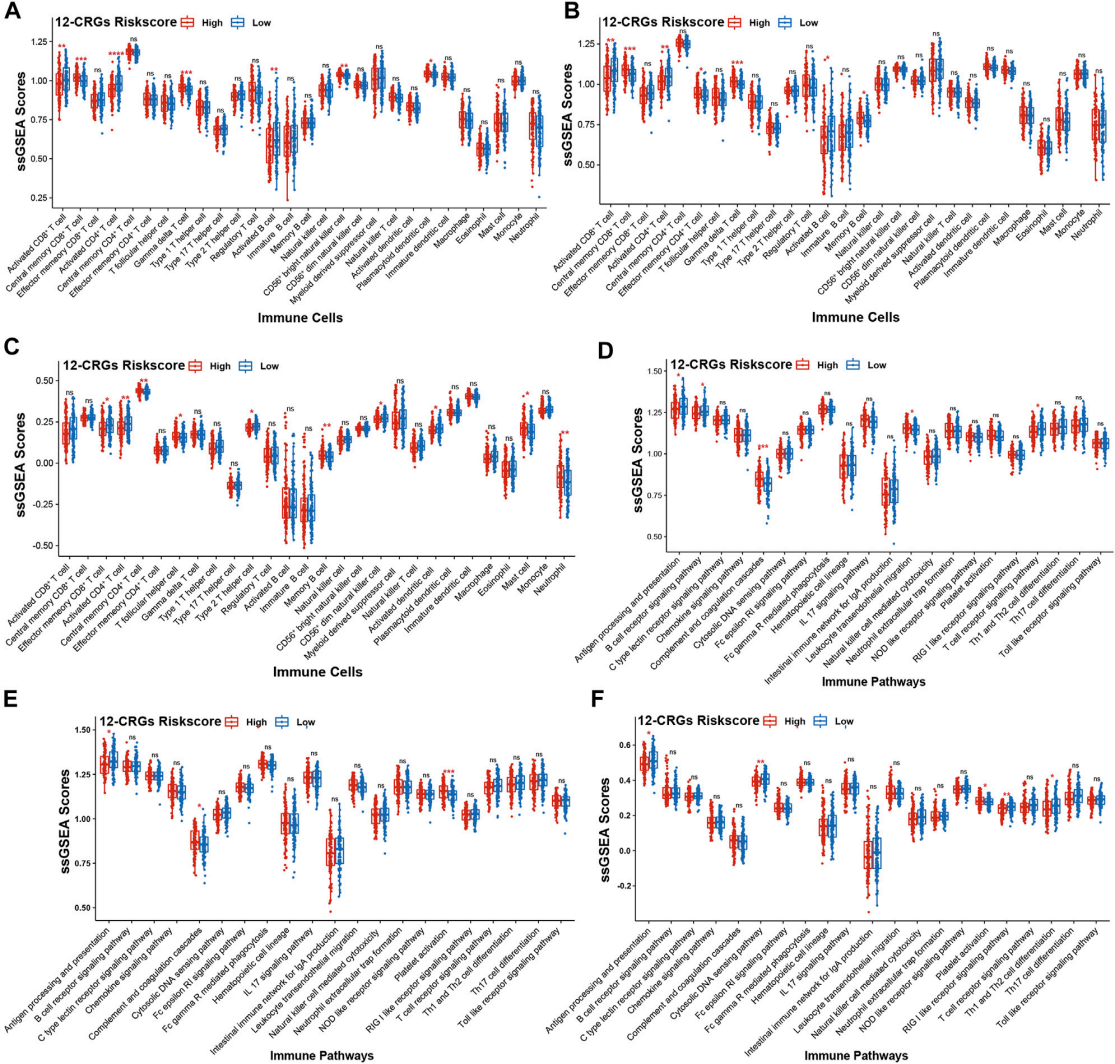
Association of the 12-CRGs signature with immune status **(A–C)** Association of the 12-CRGs signature with immune-related cells in TCGA-first **(A)**, TCGA-second **(B)** and GSE65858 **(C)** cohorts **(D–F)** Association of the 12-CRGs signature with the immune-related pathways in TCGA-first **(D)**, TCGA-second **(E)** and GSE65858 **(F)** cohorts. *p-value < 0.05; **p-value < 0.01; ***p-value < 0.001; ****p-value < 0.0001.

The interactions between cancer cells and immune cells plays an important role in regulation of cancer progression (Hara et al., 2021). To investigate the correlation between 12-CRGs risk scores and immune status in patient with HNSC, ssGSEA was used to calculate the immune scores for each patient based on 28 immune cell types and 21 immune-related pathways. In the TCGA-first and TCGA-second cohort, CD8^+^ T cell, CD4^+^ T cell and B cell immune scores were negatively correlated with high 12-CRGs risk group (Figures 6A,B). Moreover, the activation of CD4^+^ T cell was significantly suppressed in all three cohort (Figures 6A–C). Analysis of the effects of the 12-CRGs signature on the immune-related pathways revealed that antigen processing and presentation were impaired in high 12-CRGs risk group (Figures 6D–F).

### Construction and validation of nomogram for HNSC clinical utilization

**FIGURE 7:**
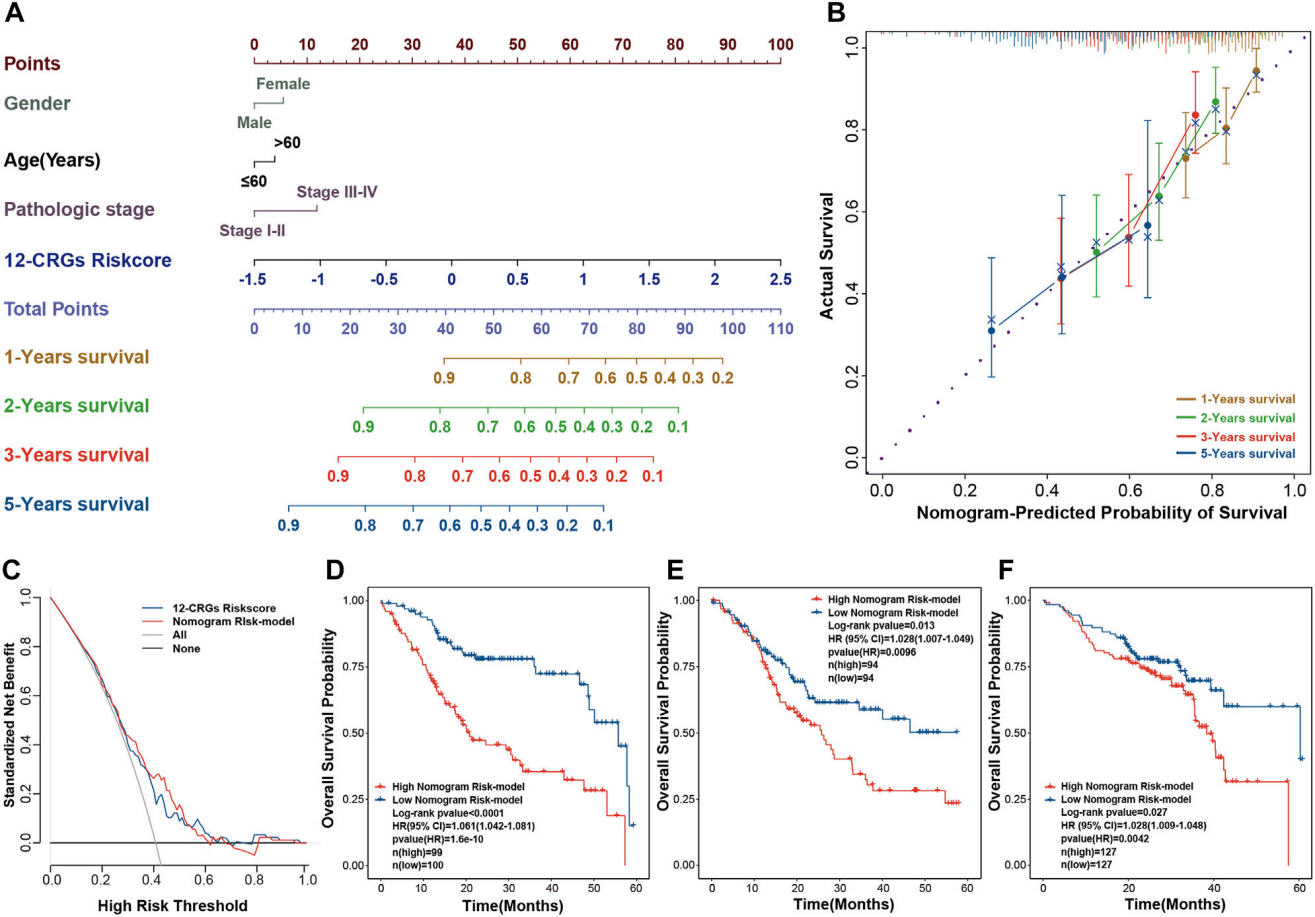
Construction of a nomogram for clinic utilization **(A)** A visual nomogram for predicting the OS at 1-, 2-, three- and 5-year after diagnosis **(B)** Calibration curve for assessing the predictive ability of the nomogram **(C)** Decision curve for evaluating the clinic use of the nomogram **(D–F)** Association of the nomogram scores with OS in TCGA-first **(D)**, TCGA-second **(E)** and GSE65858 **(F)** cohorts.

The combination of multi-genes signature and clinical features can improve the prognosis prediction of cancer patients (Yang et al., 2021). A visual nomogram that combined the 12-CRGs risk scores and clinical characters was built to predict the OS of HNSC patients (Figure 7A). Calibration curves were used to assess the predictive ability of nomogram, with the 45° line in the calibration plot represents the best prediction (Figure 7B). The nomogram scores of each HNSC patient were calculated using the nomogram, and then a nomogram risk model was developed based on nomogram scores. The decision curve verified that the nomogram risk model and 12-CRGs risk scores were clinically useful (Figure 7C). Finally, effects of nomogram scores on OS probability were also validated, and results showed that the OS probability of patients in high nomogram score was significantly decreased in all cohorts: TCGA-first (HR = 1.061 [1.042–1.081], *p*-value = 1.6e-10), TCGA-second (HR = 1.028 [1.007–1.049], *p*-value = 0.0096) and GSE65858 (HR = 1.028 [1.009–1.048], *p*-value = 0.0042) cohort (Figures 7D–F).

### Validation of genes expression in 12-CRGs signature

**FIGURE 8:**
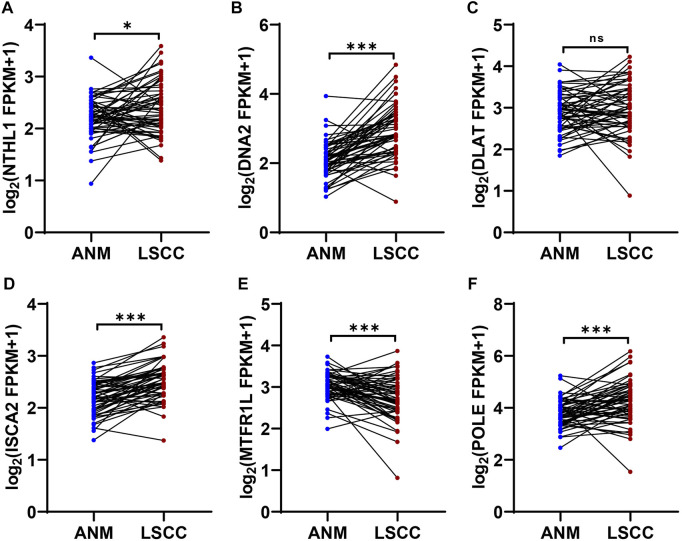
Expression validation of genes in 12-CRGs signature using RNA-Seq data of LSCC and ANM tissues **(A–F)** Expression levels of NTHL1 **(A)**, DNA2 **(B)**, DLAT **(C)**, ISCA2 **(D)**, MTFR1L **(E)** and POLE **(F)**.

In 12-CRGs signature, POLE, NTHL1, DNA2 and ISCA2 was upregulated expression in HNSC tissues while MTFR1L and DLAT was downregulated expression in HNSC tissues in both TCGA-first and TCGA-second cohort (Figures 1G, H). In order to validate the aberrant expression of POLE, NTHL1, DNA2, ISCA2, MTFR1L and DLAT in HNSC tissues, we analyzed the expression levels of these genes using RNA sequencing data of 57 LSCC and paired ANM tissues. The results showed that POLE, NTHL1, DNA2 and ISCA2 was upregulation and MTFR1L was downregulation in LSCC tissues compared to ANM tissues, however, DLAT was not statistically significant difference between LSCC and ANM tissues (Figure 8A–F).

## Discussion

To the best of our knowledge, there were no reports about the analysis of the potential correlation between CRGs and progression of HNSC so far. In current study, we investigated the effects of CRGs on HNSC development, examined the genes variation of CRGs in HNSC, analyzed the biological functions of CRGs, explored the immune influence of CRGs on HNSC, and finally developed a 12-CRGs signature and nomogram for HNSC prognosis prediction and clinic use, respectively.

Previous studies have indicated that CRGs have potential as diagnostic, and predictive biomarkers as well as therapeutic targets of cancers in clinic (Bian et al., 2022; Tsvetkov et al., 2022). In our study, we found that eleven CRGs (POLD1, NTHL1, MTFR1, CDK5RAP1, TIMMDC1, NDUFA8, PPAT, DNA2, ISCA2, POLE, RTEL1) were upregulated in HNSC compared with normal tissues, while eight genes (NDUFC1, MTFR1L, LIAS, FDX1, DLAT, PDHB, ETFDH, IDH2) were downregulated in HNSC tissues compared with normal tissues. Particularly, overexpression of ISCA2 in HNSC was observed at the RNA and protein levels, and ISCA2 was strongly correlated with OS (HR = 1.5 [1.0–2.3], *p*-value = 0.038), PFI (HR = 1.9 [1.2–2.9], *p*-value = 0.0042) and DSS (HR = 2.1 [1.3–3.6], *p*-value = 0.0046) of HNSC. ISCA2 is required for Iron-Sulfur cluster assembly and plays an essential role in the pathogenesis of multiple mitochondrial dysfunction syndromes (Weiler et al., 2020; Lebigot et al., 2021). The inhibition of ISCA2 can significantly reduce the xenograft growth of clear cell renal cell carcinoma. Mechanistically, the suppression of ISCA2 both decreases HIF-α levels and induces ferroptosis through triggering singal pathways that does not rely on ISCA2’s role in mitochondrial [4Fe-4S] assembly (Green et al., 2022), indicating that ISCA2 may be one of the potential regulators of HNSC progression.

Given that genetic mutations are one of the direct causes of cancers (Cheek and Naxerova, 2022). We investigated the mutational status of CRGs in HNSC. Thirteen genes, including MTFR1, DNA2, ACO2, PDHA1, CDK5RAP1, RTEL1, CAT, POLD1, DLAT, POLE, ATP7B, DLST and ATP7A, had somatic mutations in both training and validation cohort. Previous study reported that POLE *p*. L424V mutation was more frequent in patients with polyposis, colorectal cancer and oligodendroglioma, and four POLD1 variants (*p*.D316H, *p*. D316G, *p*. R409W, and *p*. L474P) were identified in non-polyposis colorectal cancer families. Moreover, POLE/POLD1 variants carriers direct to associated phenotype characterized by attenuated or oligo-adenomatous colorectal polyposis, colorectal cancer, and brain tumors (Bellido et al., 2016). Furthermore, POLE/POLD1 mutations were also found to be a promising predictive biomarkers for positive Immune-checkpoint inhibitor outcomes (Wang et al., 2019). ATP7B tag SNPs rs9535828 and rs9535826 were found to be correlated with platinum resistance in Chinese Han lung cancer patients (Li et al., 2014). The recurrent variant c.1121G>A (*p*.Gly374Glu; dbSNP: rs1270341616) in TCA-cycle-related gene DLST was more frequent in pheochromocytomas and paragangliomas (Remacha et al., 2019). It has been reported that the mutations in DNA2 (Zheng et al., 2020), ACO2 (Sajnani et al., 2017), and ATP7A (Li et al., 2017) were correlated with the initiation or poor clinical outcomes of cancers. CNV is another type of genetic variation in cancer (Dixon et al., 2018). We found that the chromosomal sub-bands where FDX1 and DLAT are located had deletions, and the expression levels of FDX1 and DLAT were downregulated. The downregulation of DLAT has been associated with its chromosomal deletions in liposarcoma (Crago et al., 2012), thus suggesting that the chromosomal deletions of FDX1 and DLAT may affect their own expression levels. Gene variants of CRGs may have the potential correlations with the initiation and poor clinical outcomes of HNSC.

PPIs govern a majority of cellular pathways and processes in organism (Lenz et al., 2021). We analyzed the internal interactions amongst CRGs and constructed a PPIs network consisting of 522 CRPs that were obtained from STRING database. PPIs have a non-substitutable role in all relevant biological processes, and functional enrichment analysis is an important method to investigate the collaboration of various proteins in a signaling pathway (Zhang et al., 2021). All CRPs were utilized for functional enrichment analysis based on GO and KEGG method. We found that CRPs were mainly enriched in p53 signaling pathway, TCA cycle, cell cycle, chemical carcinogenesis, carbon metabolism in cancer, iron-sulfur cluster assembly, immune-related regulation, synthetic and repair of DNA and energy metabolism signaling pathway. The enrichment of the CRGs in the TCA cycle and iron-sulfur cluster assembly was consistent with reports from a previous study by Tsvetkov et al. (Tsvetkov et al., 2022). Components required by TCA cycle and iron-sulfur cluster assembly are mainly regulatory targets for key proteins in cuproptosis process (Li et al., 2022; Tsvetkov et al., 2022). The enrichment of CRPs in p53 signaling, chemical carcinogenesis and carbon metabolism in cancer pathways might bring the potential research perspective in the relation between cuproptosis and cancers.

Multi-gene signatures have been employed for predicting prognosis and have exhibited a significantly effective ability in classifying individuals with multiple clinical-pathological risk factors, such as total mortality, chemotherapy response, and metastasis risk (Ahluwalia et al., 2021). In our study, we constructed a 12-CRGs signature consisting of CAT, MTFR1L, OXA1L, POLE, NTHL1, DNA2, ATP7B, ISCA2, GLRX5, NDUFA1, NDUFB2, and DLAT for OS prediction of HNSC. The 12-CRGs signature had the significantly adverse effects on OS (HR = 5.3 [3.4–8.2], *p*-value = 3.4e-13) of HNSC patients. Moreover, the 12-CRGs signature also exhibited the trends of poor effect on PFI (HR = 1.6 [0.92–2.7], *p*-value = 0.096) and DSS (HR = 6.4 [3.6–11], *p*-value = 2.4e-10). Some components in 12-CRGs signature have been found to have high correlation with development and progression of cancers. DNA2 have functions as both a tumor promoter and suppressor in cancers. On the one hand, DNA2 can suppress the initiation of tumors by maintaining the genomic integrity, on the other hand, it can promote the cancer cells survival through counteracting replication stress (Zheng et al., 2020). The suppression of GLRX5 activates iron-starvation response, increase intracellular free iron and in turn results in Fenton reaction and ferroptosis. In addition, GLRX5 inhibition predisposes therapy-resistant HNSC cells to ferroptosis (Lee J et al., 2020). DLAT can catalyze the conversion of pyruvate into Acetyl CoA, promote oxidative phosphorylation, ATP generation and catabolic reactions, which are important in the development of cancer (Goh et al., 2015). In addation, CAT (Galasso et al., 2021), POLE (Bellido et al., 2016), NTHL1 (Magrin et al., 2021), ATP7B (Li et al., 2017), ISCA2 (Weiler et al., 2020; Green et al., 2022), NDUFA1 (Mamelak et al., 2005) and NDUFB2(Shan et al., 2020) have also been reported to play a key role in the development of cancers. The combination of multi-genes signature and clinical characters can improve the predictive ability of prognosis for cancer patients (Yang et al., 2021). A nomogram for clinic utilization was constructed based on the 12-CRGs signature and clinical features including gender, age and pathologic stage. The nomogram scores were significantly associated with OS of HNSC in TCGA-first (HR = 1.061 [1.042–1.081], *p*-value = 1.6e-10), TCGA-second (HR = 1.028 [1.007–1.049], *p*-value = 0.0096) and GSE65858 (HR = 1.028 [1.009–1.048], *p*-value = 0.0042) cohorts. In 12-CRGs signature, POLE, NTHL1, DNA2 and ISCA2 was upregulated while MTFR1L and DLAT was downregulated in HNSC tissues in both TCGA-first and TCGA-second cohort. Furthermore, the aberant expressions of POLE, NTHL1, DNA2, ISCA2 and MTFR1L were validated by our RNA-Seq data. These results indicated that POLE, NTHL1, DNA2, ISCA2 and MTFR1L may have greater potential for clinical applications.

Immune cell response to cancer cells plays a crucial role in the regulation of cancer progression (Hara et al., 2021). In this study, correlation analysis between the 12-CRGs signature and immune status of HNSC revealed that CD4^+^ T cell activation and antigen processing and presentation were suppressed in high risk group of 12-CRGs signature. CD4^+^ T cells are a class of T helper cells, which are strongly correlated with the biological process of antitumor, and can improve the activity of other antitumor immune cells, such as CD8^+^ T cells and macrophages (Miggelbrink et al., 2021). Antigen processing and presentation are most important events in the recognition of antigens by T cells. Moreover, specific T cell tumor antigens generated through antigen processing and presentation are potential agents in the field of cancer immunotherapy (Lee MY et al., 2020). The above results demonstrated that 12-CRGs signature might influence the progression of HNSC by regulating the immune response.

Besides, some limitations should be noted in this study. Firstly, the numbers of CRGs under investigated were still limited in this study. When more CRGs are identified in the literature, a more representative number of CRGs will be included in the study. Secondly, the CRGs risk score model was constructed and validated in public database in this study. We will govern prospective multi-center clinical data to vertify the ability of CRGs risk score model in the future. Further, the effects of 12-CRGs signature on immune status of HNSC will be further investigated at the level of molecular mechanism.

## Conclusion

We comprehensively investigated the effects of CRGs on progression of HNSC at multi-omics levels, and constructed a 12-CRGs signature and nomogram for the prediction of HNSC prognosis and clinical use, respectively. Our study demonstrated that there was significant difference in expression and genes variants of CRGs between HNSC and normal tissues. ISCA2 is a CRG chosen for further analysis and was found to be upregulated in HNSC and was closely related to the prognosis of HNSC patients. Importantly, the 12-CRGs signature and nomogram had significant effects on prognosis of HNSC patients. The 12-CRGs signature was significantly associated with suppression of CD4^+^ T cell activation and antigen processing and presentation. The significant association between the expression of CRGs and HNSC progression indicated that CRGs may potential roles as diagnostic, therapeutic and prognostics biomarkers for HNSC.

## Data Availability

The original contributions presented in the study are included in the article/Supplementary Material, further inquiries can be directed to the corresponding authors.

## Supplementary material

Supplementary Figure S1 Graphical presentation of the workflow of this study.

Supplementary Figure S2 Boxplot showing of CRGs expression in paired samples extracted from all TCGA HNSC samples.

Supplementary Figure S3 Heat map of CNVs in HNSC. **(A,B)** Heat map of CNV in TCGA-1st **(A)** and TCGA-2nd **(B)** cohorts.

Supplementary Figure S4 Effects of ISCA2 on HNSC progression in the validation cohort. **(A–C)** Kaplan-Meier curve showing the association of ISCA2 with OS **(A)**, PFI **(B)**, and DSS **(C)** over 5 years in TCGA-2nd cohort. **(D,E)** Kaplan-Meier curve showing the association of ISCA2 with OS **(D)** and progression-free survival **(E)** over 5 years in the GSE65858 cohort.

Supplementary Table S1 Clinical features of HNSC patients in current study.

Supplementary Table S2 Cuproptosis-related gene list was analyzed in current study.

Supplementary Table S3 CRPs list obtained from the STRING database.

## Abbreviations

ANM, Adjacent normal mucosa; AUC, Area under time dependent ROC curve; CRGs, Cuproptosis-related genes; CNV, Copy-number variation; DSS, Disease-specific survival; GEO, Gene expression omnibus; GO, Gene ontology; HNSC, Head and neck squamous cell carcinoma; IHC, Immunohistochemistry; KEGG, Kyoto encyclopedia of genes and genomes; LASSO, Least absolute shrinkage and selection operator; LSCC, Laryngeal squamous cell carcinoma; MAF, Mutation annotation format; OS, Overall survival; PPIs, Protein-protein interactions; PFI, Progression-free interval; PCA, Principal component analysis; ROC, Receiver operating characteristic; ssGSEA, Single sample gene set enrichment analysis; TCGA, The cancer genome atlas; TISIDB, Tumor and immune system interaction database.
