# Supplementary figures and images for: An original cuproptosis-related genes signature effectively influences the prognosis and immune status of head and neck squamous cell carcinoma
Source: Front Genet. 2023 Jan 4;13:1084206. doi: 10.3389/fgene.2022.1084206 (PMC9845781; doi:10.3389/fgene.2022.1084206)

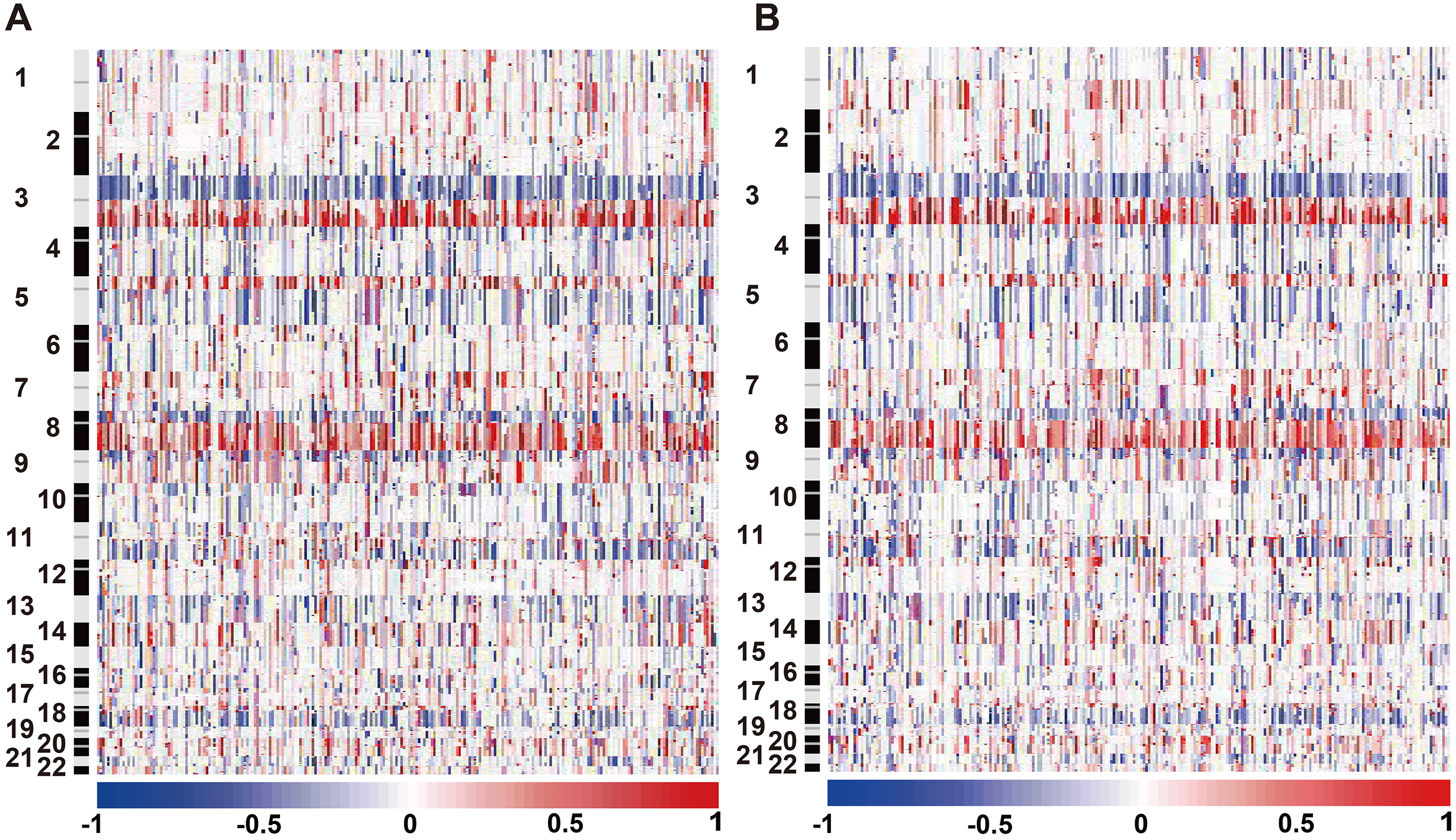

Supplement: Supplementary file 2 [file Image3.JPEG]

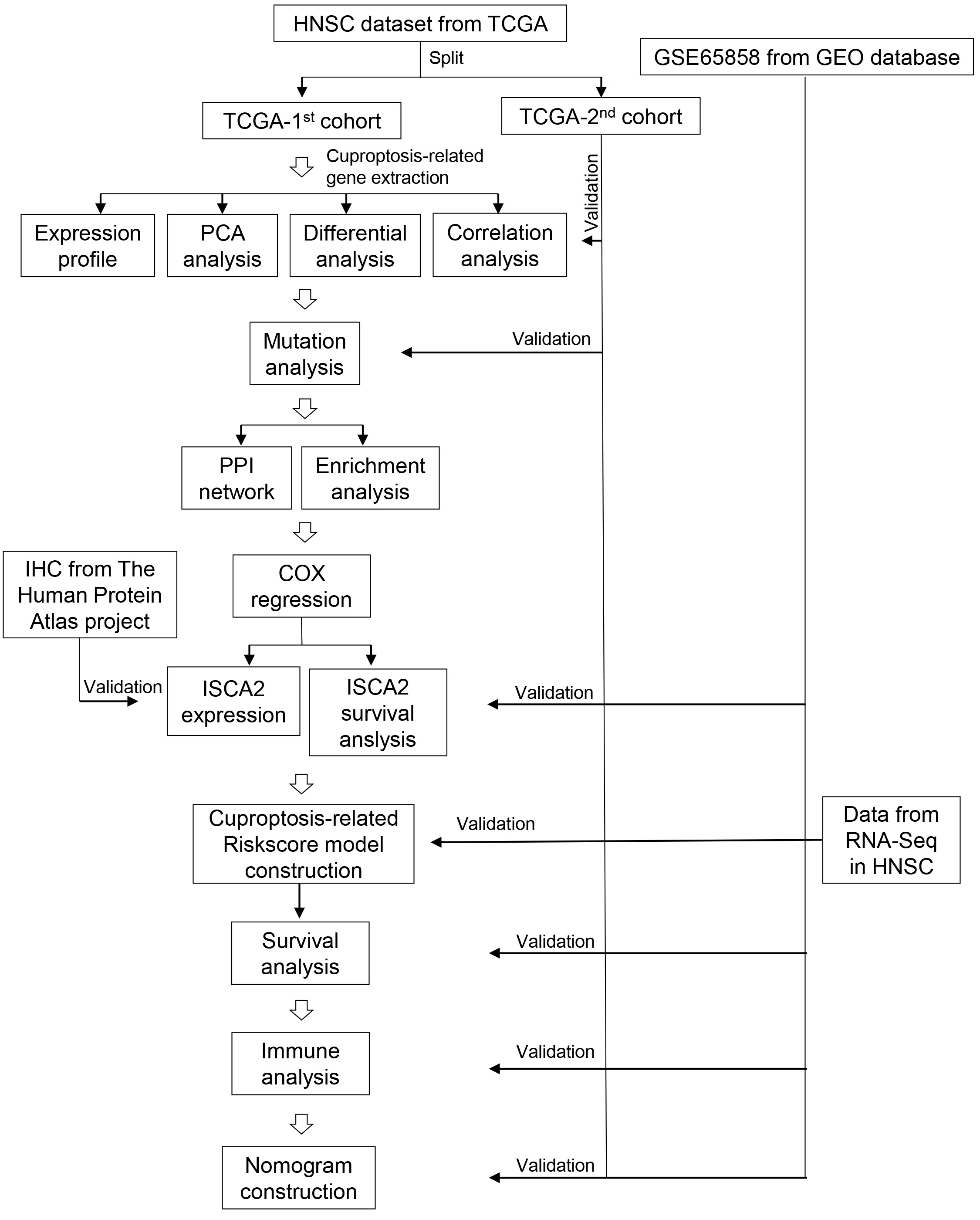

Supplement: Supplementary file 5 [file Image1.JPEG]

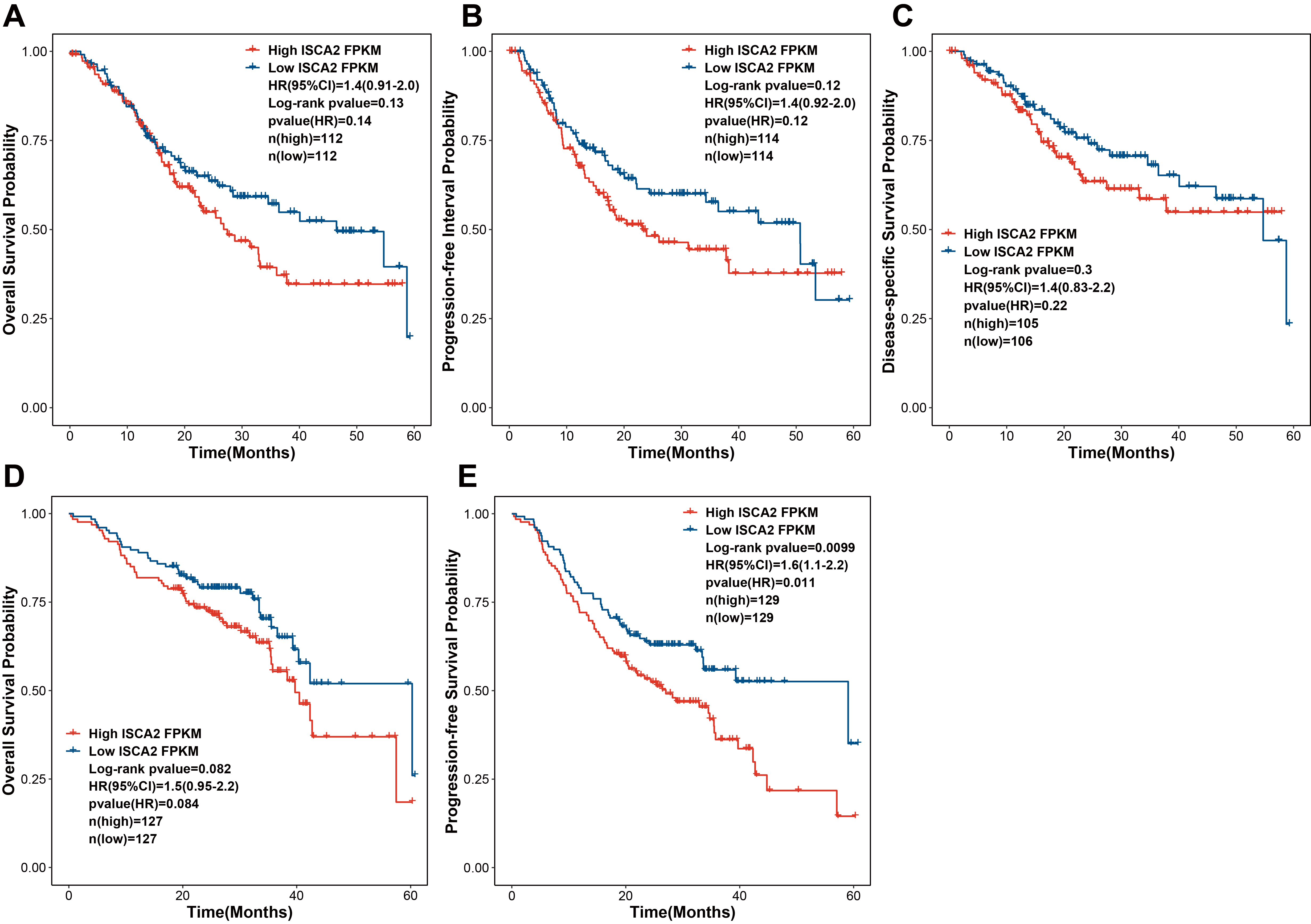

Supplement: Supplementary file 6 [file Image4.JPEG]

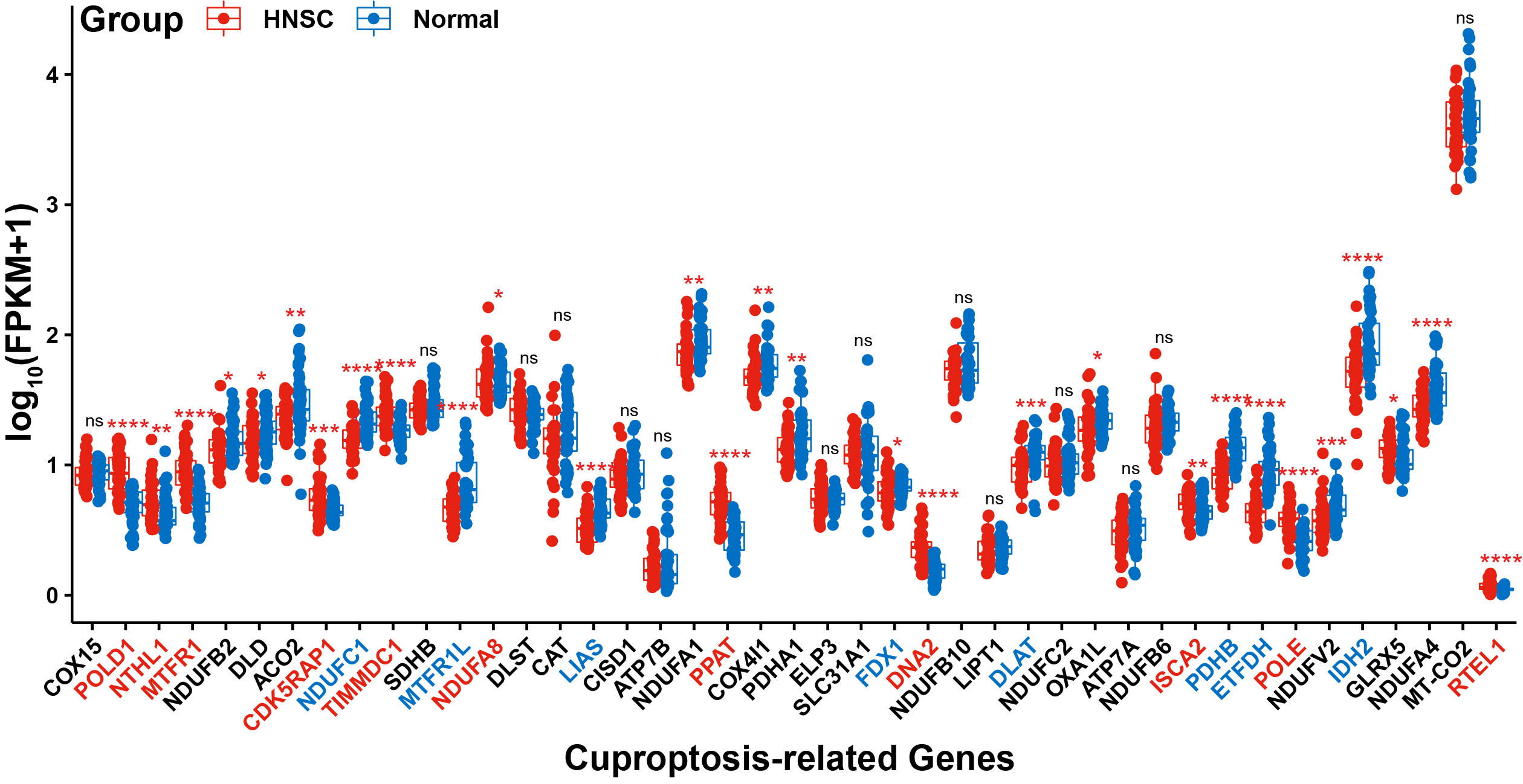

Supplement: Supplementary file 7 [file Image2.JPEG]
