# Supplementary material for: An original cuproptosis-related genes signature effectively influences the prognosis and immune status of head and neck squamous cell carcinoma
Source: Front Genet. 2023 Jan 4;13:1084206. doi: 10.3389/fgene.2022.1084206 (PMC9845781; doi:10.3389/fgene.2022.1084206)
Supplement: Supplementary file 4 [file Table1.docx]

**Supplement Table S1 Clinical features of patients with HNSC**

| **Parameters** | **Number of Cases** | | |
| --- | --- | --- | --- |
|  | **TCGA-1^st^** | **TCGA-2^nd^** | **GSE65858** |
| **Gender** |  |  |  |
| Male | 191 | 175 | **223** |
| Female | 58 | 75 | 47 |
| **Age(Years)** |  |  |  |
| ≤60 | 127 | 117 | 153 |
| >60 | 122 | 133 | 117 |
| **Pathologic T** |  |  |  |
| T0 | 1 | 0 | 0 |
| T1 | 11 | 13 | 35 |
| T2 | 40 | 40 | 80 |
| T3 | 32 | 30 | 58 |
| T4 | 43 | 56 | 97 |
| TX | 17 | 10 | 0 |
| Unknown | 105 | 101 | 0 |
| **Pathologic N** |  |  |  |
| N0 | 48 | 54 | 94 |
| N1 | 20 | 19 | 32 |
| N2 | 45 | 51 | 132 |
| N3 | 1 | 3 | 12 |
| NX | 25 | 19 | 0 |
| Unknown | 110 | 104 | 0 |
| **Pathologic M** |  |  |  |
| M0 | 42 | 56 | 263 |
| M1 | 1 | 0 | 7 |
| MX | 19 | 16 | 0 |
| Unknown | 187 | 178 | 0 |
| **Pathologic Stage** |  |  |  |
| Stage I | 10 | 15 | 18 |
| Stage II | 43 | 29 | 37 |
| Stage III | 38 | 40 | 37 |
| Stage IV | 130 | 129 | 178 |
| Unknown | 28 | 37 | 0 |
| **Alcohol History** |  |  |  |
| Yes | 92 | 95 | 239 |
| No | 48 | 51 | 31 |
| Unknown | 109 | 104 | 0 |
| **Cigarettes History** |  |  |  |
| Yes | 110 | 119 | 222 |
| No | 33 | 28 | 48 |
| Unknown | 106 | 103 | 0 |
| **HPV Testing** |  |  |  |
| Positive | 8 | 9 | 73 |
| Negative | 24 | 21 | 197 |
| Unknown | 217 | 220 | 0 |
